# Cadaver surgical training in Japan: its past, present, and ideal future perspectives

**DOI:** 10.1007/s00595-021-02330-5

**Published:** 2021-07-05

**Authors:** Toshiaki Shichinohe, Eiji Kobayashi

**Affiliations:** 1 Department of Gastroenterological Surgery II, Hokkaido University Faculty of Medicine, Sapporo, Hokkaido Japan; 2 Department of Kidney Regenerative Medicine, The Jikei University School of Medicine, 3-25-8 Nishi-Shimbashi, Minato-ku, Tokyo, 105-8461 Japan

**Keywords:** Cadaver surgical training, Body donation, Clinical anatomy

## Abstract

The framework for cadaver surgical training (CST) in Japan was established in 2012, based on the “Guidelines for Cadaver Dissection in Education and Research of Clinical Medicine” of the Japan Surgical Society (JSS) and the Japanese Association of Anatomists. Subsequently, the Ministry of Health, Labor and Welfare allocated funding from its budget for CST. By 2019, CST was being practiced in 33 medical schools and universities. Currently, the CST Promotion Committee of the JSS reviews each CST report submitted by medical schools and universities and provides guidance based on professional autonomy. This paper outlines the history of CST in Japan and presents a plan for its future. To sustain and oversee CST implementation, an operating organization, funded by stakeholders, such as government agencies, academic societies, and private companies, is needed.

## Introduction

Cadaver dissection has been the basis of medical education in anatomy since the dawn of medicine and remains the norm worldwide. Accordingly, cadaver surgical training (CST) is an effective educational method for surgeons to learn techniques [1, 2]. However, its circumstances vary by country because of religious and cultural differences in the treatment of cadavers, as well as differences in the medical education system after graduation [3]. Cadaver surgical training did not have clear implementation criteria in Japan until the “Guidelines for Cadaver Dissection in Education and Research of Clinical Medicine,” were drafted and published by the Japan Surgical Society (JSS) and Japanese Association of Anatomists (JAA) in 2012 [4]. Since the publication of these guidelines, and following budgetary measures taken by the Ministry of Health, Labor, and Welfare (MHLW), the number of medical schools and universities implementing CST has increased rapidly. In this paper, we review the history of cadaver dissection and discuss the prospects of expanding CST in Japan.

## History of CST

### History of anatomy

The first recorded cadaver dissection in Japan was performed by Doctor Toyo Yamawaki on an executed prisoner during the Edo period in 1754. In 1859, Dutch army surgeon, J. L. C. Pompe van Meerdervoort, introduced cadaver dissection as part of modern medical education in Japan. Since then, it has become one of the foundations of medical education in this country. After World War II, the body donation system was established in Japan. Modern cadaver dissection for anatomy education was conducted primarily on cadavers donated by the deceased and their families, with their consent. However, the main educational method for the post-graduate education of surgeons was on-the-job training when CST was not discussed publicly.

### Expansion of CST

Recent cadaver surgical training in North America has focused on the introduction of various implants and the mastering of techniques such as endoscopic surgery. However, in Japan, after the sensationalized newspaper coverage of a company-sponsored seminar on dental implant procedures using imported cranial cadavers in 1997, CST implementation remained stagnant for several years. Following a series of sensationalized reports, the Ministry of Health and Welfare at the time stated that improper CST implementation could lead to the crime of corpse damage, as defined by the Penal Code. Subsequently, the demand for CST implementation increased with the progress of surgical procedures and the introduction of advanced medical devices from overseas. However, CST in Japan was considered a legal “gray zone,” and was implemented by only a few universities.

## Current status of CST

### CST guidelines

Courses on CST for endoscopic surgery in each surgical field were being actively conducted at the end of the twentieth century, mainly in North America, and some Japanese doctors traveled overseas to participate in these. The increasing need for CST by Japanese surgeons became a policy issue. Thus, an MHLW research project, “Survey of Training Systems for Surgical Skills and Procedures,” was commenced in 2008 by the late Professor Satoshi Kondo, of Hokkaido University, with members of the JSS, JAA, and other surgical societies. The research group surveyed the status of surgical training including CST in Japan and worldwide, coordinated the opinions of related surgical societies, and proposed the “Draft of Guidelines for Cadaver Dissection in Education and Research of Clinical Medicine” in its third year of research.

In 2012, the JSS and the JAA jointly published the “Guidelines for Cadaver Dissection in Education and Research of Clinical Medicine” [4], based on the drafted guidelines, and with the approval of associated academic societies and the government (MHLW and the Ministry of Education, Culture, Sports, Science and Technology). These guidelines reinforce the Postmortem Examination and Corpse Preservation Act and the Body Donation Act, which forms the legal basis for CST, and allow the use of cadavers not only for CST, but also for clinical research and medical device development (Table 1). Table 2 lists the key points in the rules of the established guidelines. Cadaver surgical training can be implemented only in medical and dental schools and universities. The deceased must have consented before their death to their body being used for medical university education and research, and the family of the deceased also should have given their consent after the death of their family member. Furthermore, based on professional autonomy, the officers of each CST must report the details of the implementation and any conflicts of interest to the CST Promotion Committee of the JSS. In response to the reporting system, the JSS organized a committee to review the reports and provide advice on the proper implementation of CST to each school and university.

**Table 1:** Example of cadaver use in medical education and research [4]

(1) Acquisition of basic medical techniques Use of cadavers by doctors-in-training with the objective of acquiring the anatomical knowledge needed to perform medical techniques safely.
(2) Acquisition of basic surgical techniques and invasive techniques Use of cadavers in clinical anatomy education for learning necessary surgical and invasive techniques that can serve as alternatives for on-the-job training (OnJT) or training on animal subjects.
(3) Acquisition of surgical techniques or invasive techniques that require advanced technology Use of cadavers in clinical anatomy education and research for learning advanced surgical techniques with few OnJT opportunities or those that are difficult to learn using animals because of their anatomical differences with the human body.
(4) Research and development of new surgical techniques, invasive techniques, and medical devices Use of cadavers with the objective of researching the preclinical verification of surgical techniques or developing new surgical devices.

**Table 2:** Implementation of the use of cadavers in education and research for clinical medicine [4]

(1) The objective should be to improve medical safety through education and research for clinical medicine, and to contribute to the welfare of patients.
(2) Implementation should be done within the scope of the Postmortem Examination and Corpse Preservation Act and the Body Donation Act in medical universities (dental universities, universities with medical departments, and dental departments) for medical education and research.
(3) The cadavers used should satisfy the following conditions: Registered body donors must have provided written intent for their body to be used in medical education and research, including education through dissection by students as well as for clinical medicine, such as surgical training by doctors and dentists. Understanding and approval are also obtained from the deceased person’s family if present.
(4) Prior to implementing the training, approval should be obtained after sufficient consultation and investigation of the ethical committee of the university.

**Table 3:** Status of the use of cadavers for surgical education and research in Japan (2019)

Objectives and Fields	Cases	(%)	Details: cases (%)	
Objectives				
Education	125*	(91**)	Basic medical: technique:	33* (24**)
			Standard surgery:	86* (62**)
			Advanced surgery:	86* (62**)
Research	48*	(35**)	Clinical anatomy:	42* (30**)
			Research and development of novel surgical procedures:	13* (9*)
			Research and development of medical devices:	1* (1**)
Fields				
Orthopedic	39	(28)		
General Surgery	33	(24)	Digestive: 15 (45) Hepato-Biliary-Pancreatic: 8 (24) Respiratory: 7 (21) Cardiovascular: 1 (3) Mammary and Endocrine: 2 (6)	
Otolaryngology	17	(12)		
Brain surgery	14	(10)		
Plastic surgery	12	(9)		
Oral surgery	7	(5)		
Emergency	6	(4)		
Obstetrics	4	(3)		
Urology	4	(3)		
Anesthesiology	2	(2)		
Total	138			

*Duplicates included

**Cases/total cases

### Rapid implementation of CST

Immediately after the guidelines were published in 2013, only six of the 80 medical schools and universities in Japan reported the implementation of CST to the JSS. The MHLW supported these six universities by investing approximately 45 million yen into competitive research funds each year as operating expenses for CST from FY2013. Subsequently, presentations on CST have become more common, especially in surgical societies. CST has been recognized as an effective method for post-graduate education to promote medical safety in Japan [5–9]. In FY2018, the MHLW increased the competitive research funds for CST to approximately 300 million yen per year, with 200 million yen allocated to the acquisition of equipment and 100 million yen to operating expenses. This has continued into FY2021 with the support of the stakeholders.

**Fig. 1:**
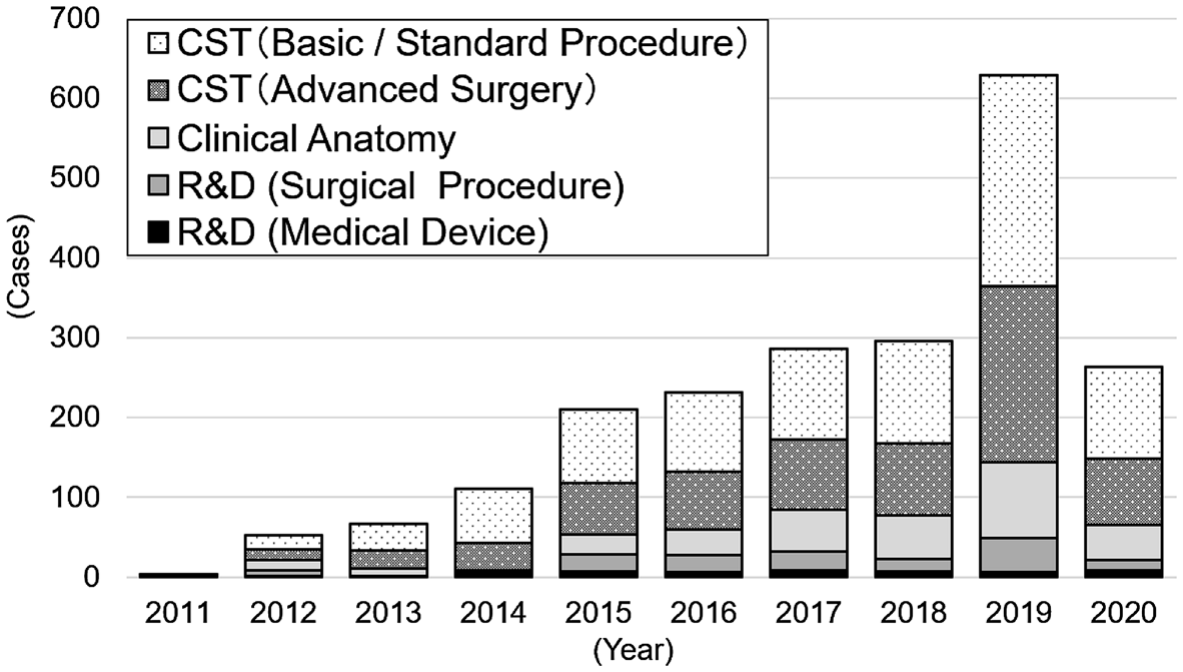
Annual trends in surgical education and research using donated cadavers in Japan. Data from the annual reports of the Japan Surgical Society CST Promotion Committee. *CST* Cadaver Surgical Training, *R&D* Research and Development

Growing government support has expediated the implementation of CST. A national report in 2019 indicated that 629 training sessions were held annually at 33 medical schools and universities, with 5042 doctors participating (Fig. 1). Furthermore, the number of donated cadavers used for CST and clinical research in Japan reached 1012 in 2019. To promote CST, many facilities in Japan use the Thiel fixation and other cadaver preservation methods for embalming rather than using fresh cadavers, which enables more endoscopic procedures such as laparoscopy [10–12].

### Diversification of needs

The widespread implementation of CST has resulted not only in an increase in the number of CST procedures reported to the JSS, but also in their diversification. When reports are categorized by purpose, programs for basic and standard surgical techniques are the most common, with those for advanced surgery also conducted to the same extent, followed by clinical anatomy programs aimed at anatomical exploration for surgery (Table 1). These results show that CST is adopted widely in the post-graduate education of doctors, from their basic training at the internship level to advanced medical research, as a safe method for learning invasive procedures without burdening patients. The implementation content in FY2019, categorized by clinical department, showed that CST was being conducted in a wide range of surgical fields, with the orthopedic specialty accounting for the largest number (28%), followed by general surgery (24%), otolaryngology (12%), and brain surgery (10%) (Table 3). Meanwhile, fewer than 10 experiments for the research and development (R&D) of medical devices have been conducted throughout this period.

## Challenges of CST

### CST in the era of COVID-19

While the number of CST sessions decreased in 2020, as a result of the impact of COVID-19, CST is still feasible even under pandemic conditions. Because the infection risks associated with on-the-job training during the COVID-19 pandemic necessitate its avoidance, CST has attracted attention as a recommended educational method. The risk of infection associated with CST is minimized by its routine use of personal protective equipment in a facility with good infection control and air ventilation. While manufacturers of Japanese medical devices have conducted verification tests of prototypes using cadavers through R&D at specialized facilities overseas, travel restrictions of the pandemic have hampered the manufacturing process. This situation further highlights the need to establish R&D platforms in Japan.

### Future of professional autonomy

To date, the promotion of CST in Japan has been headed by the JSS, an organization based on general surgery, including thoracic, gastrointestinal, pediatric, breast, and endocrine surgery. The JSS has also imposed and reviewed professional autonomy-based reports on CST in individual universities; however, the number of reports encompassing all surgical fields covered by the JSS accounts for only a quarter of recent reports (Table 3). Therefore, shifting the reviewing base to an inclusive organization that can supervise the activities of academic societies in all medical fields, such as the Japanese Medical Science Federation, is desirable.

### The future of CST

Since the CST guidelines were expanded in 2012, Japan's CST based on the body donation system has been implemented gradually, but has gained wide popularity [4]. It is noteworthy that CST in Japan has been maintained without monetary incentive in the last 10 years, whereas several other countries have implemented CST in a way that provides some benefits.

Prior to the COVID-19 pandemic, doctors from some countries with no such CST system attended expensive CST seminars with costly tuition fees, flights and accommodation. These seminars can be viewed as a “business model” among doctors seeking skill improvement, patients seeking surgery by certified doctors, and seminar organizers and organizations that arrange body donations. Japan's CST has developed without such monetary incentives, but relies on experienced surgeons who are passionate about educating surgeons eager to improve their skills. When considering that the objective of CST is to disseminate advanced medical techniques and improve medical safety, and that the wishes of body donors are also to “advance medicine”, it would be logical to develop effective incentives for the implementation of CST for both the trainer and the trainee. Examples of such incentives have been applied in a specific clinical field, such as counting CST participation as a credit for the board certifications of certain surgical specialties, and approving health insurance payment of difficult surgery when performed by surgeons with proficiency attributed to CST. In addition to the incentive for trainees, governmental and non-governmental support for clinicians, anatomists, and universities who organize CST is also important. According to Professor Rene Tolba of RWTH Aachen University, some universities in other countries evaluate CST equally to scientific publication of academic attainment.

To establish both CST and R&D infrastructure, broad discussion is necessary to build a consensus not only within the medical community, but also inclusive of the general public.
